# Research on factors influencing the performance of public health governance in the ethnic areas of Sichuan Province: a study based on the TOE framework

**DOI:** 10.3389/fpubh.2025.1525980

**Published:** 2025-10-31

**Authors:** Yuke Shi, Dandi Chen, Jing Zhou

**Affiliations:** ^1^Department of Health Policy and Management, West China School of Public Health, Sichuan University/West China Fourth Hospital, Chengdu, Sichuan, China; ^2^College of Management, Chengdu University of Traditional Chinese Medicine, Chengdu, Sichuan, China

**Keywords:** ethnic areas in Sichuan Province, TOE framework, public health governance, big data technology, synergistic governance effects

## Abstract

**Introduction:**

To better improve the performance of public health governance of the government of ethnic areas in Sichuan Province, and to promote the high-quality development of health and health care in the area.

**Methods:**

Qualitative comparative analysis of fuzzy sets and analysis of necessary conditions.

**Results:**

Big data technology, ecological environment and public opinion environment have a significant impact on the public health governance performance of ethnic area governments in Sichuan Province when they play a major role respectively; the synergistic combination of the three conditions of technology, organization and environment is conducive to the improvement of the public health governance performance of ethnic area governments in Sichuan Province.

**Suggestions:**

Develop digital economy; improve ecological environment; strengthen public opinion management; exert synergistic governance effect of technological, organizational and environmental conditions.

## 1 Introduction

In today’s society, the effectiveness of public health governance is directly related to the safety and well-being of the population. In Sichuan Province, a multi-ethnic region, due to the complex geographic environment, the relatively lagging level of economic development, and significant ethnic and cultural differences, the supply of public health services is significantly insufficient, and the capacity of governance needs to be improved. For the government, it is imperative to improve the performance of public health governance. Therefore, this study focuses on the factors influencing the performance of public health governance in the ethnic areas of Sichuan Province, based on the Technology-Organization-Environment (TOE) framework. Through an in-depth analysis of the influence of technological factors (big data technology, technological infrastructure), organizational factors (governmental attention allocation, public health resource allocation), and environmental factors (public opinion environment, ecological environment) on the performance of public health governance, we aim to reveal the underlying patterns and mechanisms. This study seeks to provide the government with insights into its inherent laws and mechanisms, enabling targeted policy recommendations and practical guidance to improve governance and promote the high-quality development of public health in these ethnic areas.

## 2 Materials and methods

### 2.1 Selection of indicators and data sources

#### 2.1.1 Selection of indicators

This study drew on the research of Chinese scholars Zhao et al. (1), and the variables and measurement indicators are shown in Table 1.

**Table 1:** Variable and measurement indicators.

Level one conditions	Level two conditions	Data measurement indicators
Outcome variable	Government performance in public health governance	Based on the concept of government performance, this study referred to Tao et al. (6) measurements of government performance in public health governance. Due to the lack of data sources, similar alternative indicators were chosen. The public health governance performance of local governments in Sichuan Province was measured using the “number of resident populations in each county” and the “successful treatment rate of active TB patients in each county” for the year 2021.
Technical condition	Big data technology	Big data technology relies heavily on infrastructure such as 5G base stations for data transmission, calculation, and analysis, and the two are inseparable. Therefore, this study measured the number of 5G base stations in each county (city and district) as of the end of 2022.
	Technology infrastructure	According to the measurement standard of Tan et al. (7), the level of technical infrastructure construction in the ethnic areas of Sichuan Province was measured by the indicator “per capita Internet broadband access” for each region in 2021.
Organizational conditions	Public health resource allocation	The number of people served by public health resources determines the efficiency of public health services. This was measured by “the ratio of the number of beds in medical institutions to the total population in each region” and “the ratio of the number of staff in public health institutions to the total population.” (8) The data were obtained from the 2021 Sichuan Statistical Yearbook and the 2021 Sichuan Health and Sanitation Statistical Yearbook.
	Allocation of government attention	Due to the unavailability of data, this study adopted indicators from other literature to measure the allocation of government attention using the ratio of government public budget input to the total population in each county (city and district) of ethnic areas in Sichuan Province in 2021.
Environmental conditions	Climate of public opinion	Referring to the measurement method of Jiang (9), this study measured the number of Weibo searches containing the keyword “epidemic situation” in counties (cities and districts) of ethnic areas in Sichuan Province from 1 January 2021 to 31 December 2023.
	Ecological environment	Urban greening construction and ecological environmental protection are inseparably related, and they complement and promote each other. In this study, we decided to use the greening coverage of each county to measure the ecological environmental conditions.

#### 2.1.2 Data sources

The data were obtained from the 2021 Sichuan Health Statistics Yearbook, the 2022 Sichuan Statistics Yearbook, the Sichuan Science and Technology Yearbook, the statistical yearbooks of the cities (states) in the ethnic areas of Sichuan Province, the websites of the people’s governments of the 60 counties (municipalities and districts) in the ethnic areas of Sichuan Province, microblogs, and other platforms.

### 2.2 Research methods

#### 2.2.1 Fuzzy-set qualitative comparative analysis (fsQCA)

Qualitative comparative analysis is based on a holistic approach to analyze complex causal problems caused by a variety of factors with the “group effect” and to explore the relationship between conditional grouping and outcome variables by comparing sample cases. Within specific categories, fuzzy-set qualitative comparative analysis (fsQCA) can better solve the problem of degree variation and local attribution, demonstrating clear advantages in solving complex antecedent issues. Therefore, it was the most suitable research method for this study.

#### 2.2.2 Necessary condition analysis (NCA)

NCA is a new method based on complex causality that can calculate the bottleneck criteria for green development more precisely. In this study, effect sizes calculated using two different estimation methods—cap envelope (CE) analysis and cap regression (CR) analysis —were used to validate condition variables in the necessary condition analysis (NCA) method. The validation followed two basic criteria: first, the effect sizes generated by the analysis of their cap functions (d) must be ⫺0.1 and second, the effect sizes from the Monte Carlo simulated permutation test must be significant.

## 3 Results

### 3.1 Data calibration

Based on the framework of fsQCA, the public health governance performance of the government in the ethnic areas of Sichuan Province was regarded as the target set, while the six variable indicators affecting this performance were regarded as the conditional set. In this process, each study sample was no longer simply attributed to a variable indicator or outcome but instead possessed an affiliation score within the corresponding set. This technique is known as calibration, where the credibility and precision of research findings are substantially enhanced by introducing external measures to align the variables with these criteria. Relying on previous research, this study deepened the understanding of the factors influencing the performance of public health governance by transforming raw data into fuzzy set affiliation scores with explanatory power through the use of the direct calibration method.

Based on the calibration criteria of Xu et al. (2), and taking into account the actual distributional characteristics of the case data, this study adopted a differentiation strategy to set the calibration anchor points. For conventional continuous variables—such as government public health governance performance, technological infrastructure, public health resources, and government attention allocation—the upper quartile (75%), median (50%), and lower quartile (25%) of the sample data were selected as the thresholds of “fully affiliated,” “intersection,” and “not affiliated at all,” respectively, with reference to the common practice in fsQCA studies within the field of public management. This quantile calibration method can effectively balance the relationship between theoretical expectations and data dispersion. Specifically, when the case data exceed the benchmark level (0.5) used in the same type of city performance assessments, the data are considered to be fully affiliated. Conversely, when the data fall below the conventional safeguard threshold (0.25), they are considered to be fully unaffiliated.

For emerging variables such as big data technology, the anchor point was constructed using the “mean ± standard deviation,” which objectively reflects the agglomeration characteristics of technological innovation factors. The thresholds for the ecological environment and public opinion environment variables were set based on the quantitative results of the policy text. Specifically, the calibrated anchor points for the ecological environment variable were “0.7” for full affiliation, “0.5” for intersection, and “0.3” for full non-affiliation. The calibration anchor point for the opinion environment variable were set as follows: “0.7” for fully affiliated, “0.3” as the crossover point, and “0.1” for full non-affiliation. The calibration details for the condition and outcome variables are shown in Table 2.

**Table 2:** Calibration of condition and outcome variables.

Level one conditions	Level two conditions	Calibration standards		
		Full affiliation	Intersection point	Completely unaffiliated
Outcome variable	Government performance in public health governance	95.3450	94.9725	91.0700
Technical condition	Big data technology	0.0825	0.0243	−0.0339
	Technology infrastructure	0.3569	0.2773	0.1681
Organizational condition	Public health resource allocation	52.6450	43.8083	31.6975
	Allocation of government attention	2625.5900	2040.0120	1237.0200
Environmental condition	Climate of public opinion	90.000	36.2000	18.1000
	Ecological environment	57.1610	48.6150	36.6970

### 3.2 Necessity analysis

As shown in Table 3, the effect size levels of the following conditions were all less than 0.1. In addition, their *p*-values were all greater than 0.05, which was not significant. Among them, the significance levels of technological infrastructure, public health resource allocation, and attention allocation were different from those of the other conditions. However, their effect sizes of 0.01 and 0.005, measured using the CE and CR methods, were less than 0.1; therefore, they cannot be considered necessary conditions for the performance of public health governance in the ethnic areas of Sichuan Province. The *p*-values of the other conditions were all 1, which was not significant. Therefore, they cannot be considered necessary conditions for the performance of public health governance in the ethnic areas of Sichuan Province.

**Table 3:** Results of NCA.

Prerequisite	Methodologies	Precision	Ceiling zone	Realm	(d)^b^	P^c^
Big data technology	CE	100%	0.000	0.78	0.000	1
	CR	100%	0.000	0.78	0.000	1
Technology infrastructure	CE	100%	0.010	1	0.010	0.167
	CR	100%	0.005	1	0.005	0.168
Public health resource allocation	CE	100%	0.010	1	0.010	0.167
	CR	100%	0.005	1	0.005	0.168
Allocation of government attention	CE	100%	0.010	1	0.010	0.167
	CR	100%	0.005	1	0.005	0.168
Climate of public opinion	CE	100%	0.000	1	0.000	1
	CR	100%	0.000	1	0.000	1
Ecological environment	CE	100%	0.000	1	0.000	1
	CR	100%	0.000	1	0.000	1

a. Calibrated fuzzy set affiliation values. b. 0.0 ≤ d < 0.1: “low level;” 0.1 ≤ d < 0.3: “medium level.” c. Permutation test in NCA (permutation test, resampling times = 10,000).

The bottleneck level measure of NCA was used to determine the minimum level that an individual antecedent condition needs to reach within its range of observations to achieve the target value. In this study, three conditions were found to have a bottleneck level for generating the results of the government’s public health governance performance in the ethnic areas of Sichuan Province—namely, technological infrastructure, allocation of public health resources, and allocation of government attention—all of which affect the outcome to roughly the same degree. Specifically, when 10 percent of public health governance performance was expected to be achieved, an input level of 0.1 percent was required for each of the three core elements: technological infrastructure, allocation of public health resources, and allocation of government attention. Furthermore, when the performance target was increased to 100 percent, the input levels of the three elements were expected to rise to 1 percent in tandem. The proportionality relationship indicated that these three elements constituted the bottleneck conditions for system enhancement. Meanwhile, big data technology, the public opinion environment, and ecological conditions did not show similar bottleneck constraint characteristics within the framework of this study.

Necessary condition analysis (NCA) focuses on necessary but insufficient individual determinants and their combinations, while qualitative comparative analysis (QCA) focuses on the combined effects of sufficient but unnecessary determinants. Therefore, this study further employed QCA to test the necessary conditions and to determine whether a single condition (including its non-sets) constitutes a necessary condition for the government’s public health governance performance. As shown in Table 4, the consistency of the necessity of the single conditions was low (all less than 0.9). This result is consistent with the findings obtained from the NCA, which showed that there are no necessary conditions for producing high levels of local government public health governance performance.

**Table 4:** Results of the NCA for individual conditions.

	High level of public health governance performance		Non-high level of public health governance performance	
Conditional variable	Consistency	Coverage	Consistency	Coverage
Big data technology	0.4059	0.66573	0.4981	0.44933
~Big data technology	0.6524	0.6568	0.6539	0.4472
Technology infrastructure	0.5665	0.5821	0.6164	0.4303
~Technology infrastructure	0.4456	0.6310	0.4013	0.3860
Public health resource allocation	0.5665	0.5821	0.6164	0.4303
~Public health resource allocation	0.4456	0.6310	0.4013	0.3860
Allocation of government attention	0.5665	0.5821	0.6164	0.4303
~Allocation of government attention	0.4456	0.6310	0.4013	0.3860
Climate of public opinion	0.6669	0.6245	0.6588	0.4190
~Climate of public opinion	0.3795	0.6209	0.4096	0.4551
Ecological environment	0.5183	0.5955	0.5406	0.4219
~Ecological environment	0.4968	0.6142	0.4817	0.4045

### 3.3 Sufficiency analysis of the conditional groupings

In the output of fsQCA, we often see three types of solutions: complex solutions, parsimonious solutions, and intermediate solutions. Among them, the intermediate solution is particularly important because it combines the advantages of the other two solutions. If a certain influencing factor appears in both the parsimonious and intermediate solutions, it is considered the core (main) condition, which has a decisive impact on the result. In this study, when setting the thresholds for the solutions, the consistency threshold was set at 0.75 and the frequency threshold was set at 1 to ensure the rigor and reliability of the study, taking into account previous studies and key elements such as sample size.

Table 5 presents the results of the grouping analysis of government public health governance based on six conditions. A total of four groupings were identified, i.e., there were four grouping paths that produced high levels of government public health governance performance in the ethnic areas of Sichuan Province. The level of consistency of both the individual solutions (groupings) and the overall solution was higher than the acceptable minimum standard of 0.75, with the consistency of the overall solution having a value of 0.8066 and the coverage of the overall solution having a value of 0.4010. It can be seen that the four groupings in Table 5 represent sufficient conditional combinations for high-level government public health governance performance.

**Table 5:** Grouping analysis of high-level government public health governance performance.

	Technology oriented+ organization support	Environment		Environment oriented + technology and organization support
Conditional configuration	Configuration 1	Configuration 2	Configuration 3	Configuration 4
Big data technology	●	⊗	⊗	⊗
Technology infrastructure	•	⊗		•
Public health resource allocation	•	⊗	⊗	•
Allocation of government attention	•	⊗	⊗	•
Climate of public opinion	⊗	⊗	●	⊗
Ecological environment	⊗	●	⊗	●
Matrix	0.7858	0.8547	0.7905	0.7990
Original coverage	0.1153	0.0836	0.1598	0.1336
Unique coverage	0.0647	0.0505	0.1363	0.0754
Consistency of solutions	0.8066			
Coverage of solutions	0.4010			

Grouping 1 indicates that the presence of big data technology plays a primary role, while technology infrastructure, public health resource allocation, and government attention allocation play a secondary role. For example, Xichang City, Liangshan Prefecture, has vigorously developed big data technology in recent years. At the same time, it built a digital economy industrial park and organized efforts to learn from global experiences in digital economy development. This city also improved technological infrastructure and gradually optimized the allocation of public health resources according to the “14th Five-Year Plan of Medical and Health Care Service System in Xichang City,” which has significantly improved the public health governance performance of the government of the region. In this grouping, when big data technology dominated, technological infrastructure, public health resource allocation, and government attention allocation played complementary roles, while other conditions were irrelevant to achieving high levels of government public health governance performance. This grouping had a consistency of 0.7858, a raw coverage value of 0.1153, and a unique coverage value of 0.0647, suggesting that this pathway was able to explain 11.53% of the cases of governmental public health governance in the ethnic areas of Sichuan Province. In addition, only 6.47% of the public health governance cases of the government in the ethnic areas of Sichuan Province could be explained by this path alone.

In grouping 2, the public opinion environment played a major role. In this study, harmonious and positive public opinion environment conditions broke the limitations of technical and organizational conditions in the area and enabled it to achieve a high level of governance performance. For example, Wenchuan County in Aba Prefecture is a city that attracts a lot of attention from the public online, and sudden epidemics and earthquakes pose major challenges for this land. As Wenchuan County attracts the attention of all sectors of society, the government enforces stricter supervision and management of the local public opinion environment, which promotes the proliferation of positive speech, strengthens the cohesion of the local community, and lays the foundation for the effective promotion of public health governance. The grouping had a consistency of 0.8547, a raw coverage value of 0.0836, and a unique coverage value of 0.0505. This indicated that the path was able to explain approximately 8.36% of the public health governance cases of the government in the ethnic areas of Sichuan Province. Moreover, only 5.05% of these cases could be explained by this path alone.

In grouping 3, the presence of an ecological environment played a major role. For example, Jiuzhaigou County in Aba Prefecture has an excellent ecological environment. It is an important ecological barrier and water conservation area in the upper reaches of the Yangtze River. This county has two national nature reserves, one national forest park, and two provincial nature reserves, and it is the ecological home to many nationally protected wild animals and plants. It has successively succeeded in creating a national “two mountains” practice innovation base, a national ecological civilization construction demonstration base, and a national ecological civilization construction model. It has been successfully established as a national “two mountains” practice innovation base and national ecological civilization construction demonstration county. The favorable ecological environment in the area prevents and reduces the spread of diseases, ensures the quality of life for residents, and reduces the difficulty while improving the efficiency of the government’s public health management efforts. The group pattern had a consistency of 0.7905, a raw coverage value of 0.1598, and a unique coverage value of 0.1363. It indicates that the pathway was able to explain approximately 15.98% of the governmental public health governance cases in the ethnic areas of Sichuan Province. Meanwhile, only 13.63% of the governmental public health governance cases could be explained by this path alone.

In grouping 4, the public opinion environment played a primary role, and the presence of technological infrastructure, public health resource allocation, and government attention allocation played a secondary role. For example, the government issued the “Basic Public Health Service Program” and the “Basic Medical Care Performance Evaluation Program” in 2023, which supervise primary healthcare institutions, health service centers, and township health centers that provide basic public health services and basic medical care in the whole county. The programs constantly rectify technological infrastructure and optimize the allocation of public health resources. At the same time, they actively give feedback to the public, which makes the public opinion environment open and transparent and promotes the performance of public health governance. The grouping had a consistency of 0.799, a raw coverage value of 0.1336, and a unique coverage value of 0.754. This pathway was able to explain approximately 13.36% of the public health governance cases in the ethnic areas in Sichuan Province. In addition, only 7.54% of the government public health governance cases in the ethnic areas of Sichuan Province could be explained by this path alone.

## 4 Conclusion and recommendations

### 4.1 Research conclusion

4.1.1 Big data technology, the ecological environment, and the public opinion environment have a significant impact on the performance of the government’s public health governance in the ethnic areas of Sichuan Province when they play a major role individually.

The application of big data is associated with the reduction of government information collection and exchange costs, and its fast and accurate information acquisition characteristics can support the improvement of government public health governance. In addition, people living in an environment with fresh air and high vegetation coverage experience better quality of life and a lower risk of illness, which, in turn, reduces the difficulty faced by the government in public health governance. At the same time, in the age of information and technology, positive public opinion guidance can increase public awareness of public health events and encourage citizens to offer suggestions to the government, thereby motivating the government to strengthen public health management. 4.1.2 The synergistic combination of the three conditions of technology, organization, and environment is conducive to the improvement of the performance of the government’s public health governance in the ethnic areas of Sichuan Province.

Based on the sufficiency analysis of the conditional groupings, the resulting four groupings can be divided into three governance modes overall: technology-based + organization-based, environment-based, and environment-based + technology and organization-based. Two of these modes function as combinations of multiple conditions. In governance mode one (technology-based + organization-based), technology and organization work in synergy, and the cooperation between big data technology applications and governmental actors facilitates the collection and analysis of information, improves the efficiency of governance, and enables the government to effectively carry out public health governance. Governance mode three (environment-based + technology and organization-based) reflects the synergy among technology, environment, and organization. These three conditions work in synergy. On the basis of a good environment, the role of both technical and organizational conditions can effectively improve public health governance.

### 4.2 Policy recommendations

#### 4.2.1 Develop a digital economy and expand the application of big data technology

Due to the sparse population and limited resources in ethnic areas, there is an imbalance in the distribution of resource allocation. The application of big data technology can better identify the differentiated needs of different regions, enable the rational allocation of resources, and effectively alleviate the shortage of resources in ethnic areas. At the same time, ethnic areas should accelerate the development of big data platforms for early warning and analysis systems. These platforms should collect and analyze information on potential public health emergencies through multiple channels and, in coordination with the supply and demand dynamics of medical resources, enable timely monitoring and early warnings—ensuring adequate preparation before peak demand periods (3).

#### 4.2.2 Improve the ecological environment and create a beautiful and healthy city

The ecological environment is an important element of environmental conditions, including the rational use and protection of natural resources such as forests, lakes, and grasslands. In the context of public health governance in the ethnic areas of Sichuan Province, a good ecological environment is conducive to improving the quality of life of residents and reducing the spread of diseases. The government of ethnic areas should further establish good ecological advantages, create clean and hygienic urban environments, reduce the rate of disease transmission, avoid the frequent occurrence of sudden public health events, and lay the foundation for the development of high-quality public health governance.

#### 4.2.3 Strengthen public opinion management and build a harmonious public opinion environment

Since the 21st century, public health events such as SARS, influenza, and epidemics have occurred frequently, which not only affect the normal life pattern of society but also have an immeasurable impact on the public’s psychological well-being. Whether the government can react quickly and guide public opinion scientifically in the face of public health emergencies has increasingly become an important indicator for judging its performance in public health governance. Therefore, the government of ethnic areas in Sichuan Province should actively address health emergencies. The media should utilize big data to increase the visibility and credibility of official information releases, guide public attitudes and create a favorable public opinion environment.

#### 4.2.4 Integration of regional technical, organizational, and environmental conditions to achieve a synergistic governance effect

Based on the case data from 60 counties (cities and districts) in the ethnic areas of Sichuan Province, it can be seen that only policies that are tailored to local conditions and in line with the actual stage of development can solve the problems and challenges faced in the field of public health governance. At the same time, the use of big data technology, do a good job of health emergency data and information and monitoring and early warning platform docking, information sharing as the main engine to promote the main synergies, connectivity, strengthen the medical and defense information synergy, so as to increase the information sharing between the main body, and to establish a cross-sectoral integrated epidemic prevention and control information platform (5), in order to promote the synergistic combination of the three technology, organization and the environment, to improve the performance of the government’s public health governance. Specifically, municipal departments can be used as guides to implement “one-to-one” financial guidance and coordination. They can jointly formulate plans for the use of funds and, based on the principle of scientific coordination and needs-based allocation, rationally coordinate the input of financial resources for public health. They can reasonably direct financial funds to remote ethnic minority areas according to need, maximizing the effectiveness of public health funding. This aims to maximize the effectiveness of financial resources for public health services and promote technological development and environmental construction in ethnic minority areas, thereby fully leveraging the synergistic effects of various elements of governance.

Although this study strived to be rigorous, the following methodological limitations should be interpreted with caution. First, some core concepts were measured using proxy indicators, such as tuberculosis treatment rate and resident population size, which, although validated in the literature, may still fail to capture the multidimensional features of governance performance. Second, the timeliness and measurement errors in government yearbooks and social media data might have affected the calibration accuracy, although this was mitigated through cross-validation using multiple sources. Finally, although the group analysis based on the cross-sectional data could identify synergistic associations among the variables, it could not conclusively prove causal mechanisms, suggesting that the findings should be treated as exploratory findings rather than deterministic laws. It is recommended that follow-up studies incorporate tracking data and process-tracing methods to further test the dynamic causal pathways underlying improvements in governance effectiveness.

## Data Availability

The original contributions presented in the study are included in the article/supplementary material, further inquiries can be directed to the corresponding authors.
